# Supplementary material for: Exploring the psycho-social burden of infertility: Perspectives of infertile couples in Cape Coast, Ghana
Source: PLoS One. 2024 Jan 25;19(1):e0297428. doi: 10.1371/journal.pone.0297428 (PMC10810504; doi:10.1371/journal.pone.0297428)
Supplement: Attachment — Submitted filename: Response to Reviewers.docx [file pone.0297428.s002.docx]

PONE-D-23-28365

Exploring the psycho-social burden of infertility: Perspectives of infertile couples in Cape Coast, Ghana

| **Reviewers Comments. Reviewer #1** | **Reactions to the Comments** |
| --- | --- |
| 1. In the abstract where it says, “we explored the impact of infertility on the psychological and social health of infertile couples” I would add “receiving fertility treatment”. Similarly, in other relevant places of the manuscript, it should be highlighted that the targeted population was not general infertility couples but the ones attending fertility clinics. | Thanks for the comment. This has been done. |
| 2. The Social Ecological Model (SEM) - can the author explain better the difference between interpersonal, organizational and community please? as at the moment they seem to overlap. | We are grateful for this comment. It was an oversight on our part. This has been corrected. See pages 4 and 5. |
| 3. Can the authors add whether fertility treatment in Ghana is supported by the National health system or whether the affected couples need to pay for treatment as this would clarify better the socio-economic status of the interviewed couples. | This information has been added. See page 4. |
| 4. Can the author expand on further bias in the selected population, in the discussion section. For example, that these people were proactively addressing the infertile issue and were willing (and able) to seek help. Would this attitude come from a more or less ‘desperate’ population? And how easy/difficult would it be to recruit a more general infertile population? And what are the implications on the results of this study, by using this restricted population? | This has been provided in the discussion section. See page 19. |
| 5. Page 15 - “This strategy works better when the husbands of the various couples are very supportive of their wives”. This statement seems to highlight that women need support from men but not the other way around when the cause of infertility is with the man. Can the authors expand a bit on this in the discussion section? is it a preconception that women need a husbands’ support as they are considered the main cause of infertility but not the other way around? | Thank you for this comment. We have expanded on this in the discussion section. Please see page 20, paragraph 2 for the addition. |
| 6. Previous literature has reported the difference in perception and attitude towards infertility between developing and developed countries. Can the authors discuss this and if the study confirmed any of these differences? | While we acknowledged that these differences exist in the literature, we do not wish to go in that direction as this is a qualitative study and we want to limit the discussion within context. |
| 7. In the conclusion, I think one important message, other than educating interpersonal-level actors, is also for the health care professionals to provide more comprehensive care (including psychological) to the affected couples. | We are grateful for this comment. We have included this suggestion as it is very important for the study. See the conclusion section. |
| 8. Table 2 - can the authors add the Frequency of sub-theme in an added column please? | Thanks for this comment. However, given the focus of this paper we do not wish to quantify the issues. |
| 9. page 11- “self-distractive” did the authors mean “self-destructive”? | This has been corrected. It now reads “Self-destructive”. |
| 10. Page 20 - “although the source of stigma and coping strategies” I suggest deleting the text “and coping strategies” to make sense of the phrase. | Thank you for this observation. The text has been deleted. See page 20. |
|  |  |
| **Reviewer Comments: Reviewer #2** | **Reactions to the comments** |
| 1. Aim of the study not clearly identified; this needs to be specified in the study background. | Thank you for the comment. However, the aim of the study was stated in page 4 at the end of the introduction section. |
| 2. Methods – it is not specified which phenomenological approach was used for the study – descriptive or interpretive with clear descriptions of its respective philosophical underpinnings and how that was used as guide for the data collection and analysis. The research design fits an exploratory-descriptive study. | We are grateful for this comment. This information has been provided. Please see page 5, under “Materials and Methods” section. |
| 3. Minor phrase correction Table 2: Themes and subthemes from the infertile couples; Subthemes – need to be numbered. | Thank you for this observation. The corrections have been made. |
| 4. In the data analysis, how were the themes and subthemes generated noting that a model was used for the research? How was the Social Ecological Model (SEM) used as the organizing framework for the research noting that study themes are largely predetermined when models/frameworks are used in research; it is unclear how the constructs of the existing model guided the development of the current themes/subthemes which are different from the constructs of the model. | We are grateful for this observation. Further information has been provided on how the themes and subthemes were generated. See page 7 paragraph 2 and 3. |
| 5. Explanatory/analytic statement have been provided for some quotes but not all; these transition statements need to be provided between participant quotes before moving on to other themes and subthemes and subsequent headings. | Explanatory/analytic statements have now been provided for the rest of the quotes. |
| 6. Study limitations - Explain measures to address language translation implications on the findings of the study, its inherent dynamics, how it influences participants' narratives and the meanings of the text. It is important to note potentially lost meanings of the translated text since the interviews were conducted in the local language and translated into the English language. Within the Ghanaian local language, there are words or phrases or expressions that do not have its direct translations into English. | This has been addressed in page 6. |
| 7. Minor sentence correction - The authors express appreciation to the management of the study health facilities and the study participants shared who their perspectives with us. | We are grateful for this observation. The sentence has been corrected. |
