# Supplementary material for: Exploring the psycho-social burden of infertility: Perspectives of infertile couples in Cape Coast, Ghana
Source: PLoS One. 2024 Jan 25;19(1):e0297428. doi: 10.1371/journal.pone.0297428 (PMC10810504; doi:10.1371/journal.pone.0297428)
Supplement: Attachment — Submitted filename: Response to Reviewers.docx [file pone.0297428.s003.docx]

PONE-D-23-28365R1

Exploring the psycho-social burden of infertility: Perspectives of infertile couples in Cape Coast, Ghana

| **Reviewers Comments. Reviewer #1** | **Reactions to the Comments** |
| --- | --- |
| 1. “My only comment is on the edited text on page 19, paragraph 2, about participants being infertile couples who were actively seeking treatment. I think that couples who have resigned to fate might have less adverse psychological impact than the ones seeking treatment, however the blocker to treatment access could also be financial. Couples that cannot afford to pay for the treatment could have a more severe psychological impact than the ones able to pay for it. I suggest the authors add this potential option too”. | We are grateful to the reviewer for this minor but important comment. We have included this in the revised manuscript. |
| 2. There is typo on page 20, 2nd paragraph, edited text - “cooing strategy” should be “coping strategy”. | The typo has been corrected. |
