# Supplementary material for: Exploring the psycho-social burden of infertility: Perspectives of infertile couples in Cape Coast, Ghana
Source: PLoS One. 2024 Jan 25;19(1):e0297428. doi: 10.1371/journal.pone.0297428 (PMC10810504; doi:10.1371/journal.pone.0297428)
Supplement: Attachment — Submitted filename: Reviewer Comments - Manuscript Number PONE-D-23-28365.pdf [file pone.0297428.s001.pdf]

Upload Reviewer Attachments for Manuscript Number PONE-D-23-28365
"Exploring the psycho-social burden of infertility: Perspectives of infertile couples in Cape Coast, Ghana"

- Aim of the study not clearly identified; this needs to be specified in the study background.

- Methods – it is not specified which phenomenological approach was used for the study – descriptive or interpretive with clear descriptions of its respective philosophical underpinnings and how that was used as guide for the data collection and analysis. The research design fits an exploratory-descriptive study

- Minor phrase correction Table 2: Themes and subthemes from the infertile couples; Subthemes – need to be numbered.

-In the data analysis, how were the themes and subthemes generated noting that a model was used for the research? How was the Social Ecological Model (SEM) used as the organizing framework for the research noting that study themes are largely predetermined when models/frameworks are used in research; it is unclear how the constructs of the existing model guided the development of the current themes/subthemes which are different from the constructs of the model.

- Explanatory/analytic statement have been provided for some quotes but not all; these transition statements need to be provided between participant quotes before moving on to other themes and subthemes and subsequent headings.

-Study limitations - Explain measures to address language translation implications on the findings of the study, its inherent dynamics, how it influences participants' narratives and the meanings of the text. It is important to note potentially lost meanings of the translated text since the interviews were conducted in the local language and translated into the English language. Within the Ghanaian local language, there are words or phrases or expressions that do not have its direct translations into English.

- Minor sentence correction - The authors express appreciation to the management of the study health facilities and the study participants shared who their perspectives with us.
